# Handshape Recognition Using Skeletal Data

**DOI:** 10.3390/s18082577

**Published:** 2018-08-06

**Authors:** Tomasz Kapuscinski, Patryk Organisciak

**Affiliations:** Department of Computer and Control Engineering, Rzeszow University of Technology, 35-959 Rzeszow, Poland; patrykorganisciak@gmail.com

**Keywords:** handshape recognition, sign language, finger alphabet, skeletal data

## Abstract

In this paper, a method of handshapes recognition based on skeletal data is described. A new feature vector is proposed. It encodes the relative differences between vectors associated with the pointing directions of the particular fingers and the palm normal. Different classifiers are tested on the demanding dataset, containing 48 handshapes performed 500 times by five users. Two different sensor configurations and significant variation in the hand rotation are considered. The late fusion at the decision level of individual models, as well as a comparative study carried out on a publicly available dataset, are also included.

## 1. Introduction

Handshapes are the basis of so-called finger alphabets that are used by deaf people to express words for which there are no separate signs in sign languages. The same handshapes, shown for various positions and orientations of the hand, are also important components of dynamic signs occurring in sign languages. Moreover, in the case of the so-called minimal pairs, the shape of the hand is the only distinguishing feature. Therefore, building a complete system for automatic recognition of the manual part of sign language is not possible without solving the problem of recognizing static handshapes.

The problem is challenging. Handshapes occurring in finger alphabets are complicated. A projection, that takes place during the image formation in a camera, results in significant loss of information. Individual fingers overlap each other or remain completely covered. In addition, some handshapes are very similar. Moreover, a movement trajectory is not available and therefore a detailed analysis of the shape is required. In the case of typical cameras, including stereo cameras, a big challenge is a dependence on variable backgrounds and lighting conditions. Individual differences in showing particular shapes by different users need to be considered as well. Therefore, systems developed in a controlled and sterile laboratory environment do not always work in demanding real-world conditions.

Currently, there are imaging devices on the market which operate both in the visible and near-infrared and provide accurate and reliable 3D information in the form of point clouds. These clouds can be further processed to extract skeletal information. An example of such a device is the popular Kinect controller, which, along with the included software, provides the skeletal data for the entire body of the observed person. There are similar solutions, with smaller observation area and higher resolution, for obtaining skeletal data for the observed hand. Examples of such devices are some time-of-flight cameras or a Leap Motion controller (LMC). These are in early stages of development but technological progress in this area is fast. For example, the first version of the Leap Motion Software Development Kit (SDK) was able to track only visible parts of the hand, but the version 2 uses some prediction algorithms, and the individual joints of each finger are tracked even when the controller cannot see them. It is expected that sooner or later, newer solutions will emerge. Therefore, it is reasonable to undertake research on the handshape recognition based on skeletal data.

Despite a number of works in this field, the problem remains unresolved. Current works are either dedicated to one device only or deal with a few simple static shapes or dynamic gestures, for which the great support is the distinctive role of the motion trajectory.

In this paper, a method of handshapes recognition, based on skeletal data is described. The proposed feature vector encodes the relative differences between vectors associated with the pointing directions of the fingers and the palm normal. Different classifiers are tested on the demanding dataset, containing 48 handshapes performed by five users. Each shape is repeated 500 times by each user. Two different sensor configurations and significant variation in the hand rotation are considered. The late fusion at the decision level of individual models, as well as comparative study carried out on a publicly available dataset, are also included.

The remainder of this paper is organized as follows. The recent works are characterized in Section 2, Section 3 describes the method, Section 4 discusses the experiment results, and Section 5 summarizes the paper. Appendix A contains the full versions of the tables with the results of leave-one-person-out cross-validation.

## 2. Recent Works

The suitability of the skeletal data, obtained from the LMC, for Australian Sign Language (Auslan) recognition has been explored in [1]. Testing showed that despite the problems with accurate tracking of fingers, especially when the hand is perpendicular to the controller, there is a potential for the use of the skeletal data, after some further improvement of the provided API.

An extensive evaluation of the quality of skeletal data, obtained from the LMC, was also tested in [2]. Static and dynamic measurements were performed using a high-precision motion tracking system. For static objects, the 3D position estimation with the standard deviation less than 0.5 mm was reported. A spatial dependency of the controller’s precision was also tested. In [1,2] the early version of the provided software was used. Recently, the stability of tracking has been significantly improved.

In [3], the skeletal data was used to recognize a subset of 10 letters from American Manual Alphabet. Handshapes were presented 10 times by 14 users. The feature vector was based on the positions and orientations of the fingers measured by the LMC. The multi-class support vector machine (SVM) classifier was used. The recognition accuracy was 80.86%. When the feature vector was augmented by features calculated from the depth map obtained with the Kinect sensor, the recognition accuracy increased to 91.28%.

In [4], the 26 letters of the English alphabet in American Sign Language (ASL) performed by two users were recognized using the features derived from the skeletal data. The recognition rate was 72.78% for the k-nearest neighbor (kNN) classifier and 79.83% for SVM.

Twenty-eight signs corresponding to the Arabic alphabet, performed 100 times by one person were recognized using 12 selected attributes of the hand skeletal data [5]. For the Naive Bayes (NB) classifier, the recognition rate was 98.3% and for the Multilayer Perceptron (MP) 99.1%.

In [6], the 50 dynamic gestures from Arabic Sign Language (ArSL), performed by two persons, were recognized using the feature vector composed of positions of fingers and distances between them and multi-layer perceptron neural network. The recognition accuracy was 88%.

A real-time multi-sensor system for ASL recognition was presented in [7]. The skeletal data, collected from Leap Motion sensors, was fused using multiple sensors data fusion and the classification was performed using hidden Markov models (HMM). The 10 gestures, corresponding to the digits from 0 to 9, were performed by eight subjects. The recognition accuracy was 93.14%.

In [8], the 24 letters from ASL were recognized using the feature vector that consists of the normal vector of the palm, coordinates of fingertips and finger bones, the arm direction vector, and the fingertip direction vector. These features were derived from the skeletal data provided by LMC. The decision tree (DT) and genetic algorithm (GA) were used as the classifier. The recognition accuracy was 82.71%.

Five simple handshapes were used to control a robotic wheelchair in [9]. Skeletal data was acquired by LMC. Feature vector consisted of the palm roll, pitch and yaw angles, and the palm normal direction vector. Block Sparse Representation (BSR) based classification was applied. According to the authors, the method yields accurate results but no detailed information about experiments and obtained recognition accuracy are given.

In [10], 10 handshapes corresponding to the digits in Indian Sign Language were recognized. The feature vector consisted of the distances between the consecutive fingertips and palm center and the distances between the fingertips. The features were derived from skeletal data acquired by LMC. Multi-Layer Perceptron (MP) neural network with back propagation algorithm was used. Each shape was presented by four subjects. The recognition accuracy of 100% is reported in the paper.

In [11], 28 letters of the Arabic Sign Language were recognized using the body and hand skeletal data acquired by Kinect sensor and LMC. One thousand four hundred samples were recorded by 20 subjects. One hundred and three features for each letter were reduced to 36 using the Principal Component Analysis algorithm. For the SVM classifier, the recognition accuracy of 86% is reported.

In [12], 25 dynamic gestures from Indian Sign Language were recognized using a multi-sensor fusion framework. Data was acquired using jointly calibrated Kinect sensor and LMC. Each word was repeated eight times by 10 subjects. Different data fusion schemes were tested and the best recognition accuracy of 90.80% was reported for the Coupled Hidden Markov Models (CHMM).

Twenty-eight handshapes corresponding to the letters of the Arabic alphabet were recognized using skeletal data from LMC and RGB image from Kinect sensor [13]. Gestures were performed at least two times by four users. Twenty-two of 28 letters were recognized with 100% accuracy.

In [14], Rule Based-Backpropagation Genetic Algorithm Neural Network (RB-BGANN) was used to recognize 26 handshapes corresponding to the alphabet in Sign System of Indonesian Language. Thirty-four features, related to the fingertips positions and orientations, taken from the hand skeletal data acquired by LMC, were used. Each gesture was performed five times. The recognition accuracy was 93.8%.

The skeletal data provided by the hand tracking devices LMC and Intel RealSense was used for recognizing 20 of the 26 letters from ASL [15]. The SVM classifier was used. The developed system was evaluated with over 50 individuals, and the recognition accuracy for particular letters was in the range of 60–100%.

In [16], a method to recognize static sign language gestures, corresponding to 26 American alphabet letters and 10 digits, performed by 10 users, was presented. The skeletal data acquired by LMC was used. Two variants of the feature vector were considered: (i) the distances between fingertips and the center of the palm, and (ii) the distances between the adjacent fingertips. The nearest neighbor classifier with four different similarity measures (Euclidean, Cosine, Jaccard, and Dice) was used. The obtained recognition accuracy varied from 70–100% for letters and 95–100% for digits.

Forty-four letters of Thai Sign Language were recognized using the skeletal data acquired by LMC and the decision trees [17]. The recognition accuracy of 72.83% was reported, but the authors do not indicate how many people performed gestures.

In [18], the skeletal data, acquired from two Leap Motion controllers, was used to recognize 28 letters from Arabic Sign Language. Handshapes were presented 10 times by one user. For the data fusion at features level and Linear Discriminant Analysis (LDA) classifier, the average accuracy was about 97.7%, while for classifier level fusion using Dempster-Shafer theory of evidence—97.1%.

Ten static gestures performed 20 times by 13 individuals were recognized using the new feature called Fingertips Tip Distance, derived from LMC skeletal data, and Histogram of Oriented Gradients (HOG), calculated from undistorted, raw sensor images [19]. After dimension reduction, based on Principal Component Analysis (PCA), and feature weighted fusion, the multiclass SVM classifier was used. Several variants of feature fusion were explored. The best recognition accuracy was 99.42%.

In [20], 28 isolated manual signs and 28 finger-spelling words, performed four times by 10 users, were recognized. The proposed feature vector consisted of fingertip positions and orientations derived from the skeletal data obtained with LMC. The SVM classifier was used to differentiate between manual and finger spelling sequences and the Bidirectional Long Short-Term Memory (BLSTM) recurrent neural networks were used for manual sign and fingerspelled letters recognition. The obtained recognition accuracy was 63.57%.

Eight handshapes, that can be used to make orders in a bar, were recognized in [21]. Each shape was presented three times by 20 participants. The feature vector consisted of normalized distances between the tips of the fingers and the center of the palm and was calculated from row skeletal data provided by LMC. Three classification methods: kNN, MP and Multinomial Logistic Regression (MLR) were considered. The best recognition accuracy of 95% was obtained for kNN classifier.

In [22], fingertip distances, fingertip inter-distances, and hand direction, derived from skeletal data acquired by LMC as well as the RGB-D data provided by Kinect sensor were used for sign language recognition in a multimodal system. Ten handshapes, performed 10 times by 14 users were recognized using data-level, feature-level, and decision-level multimodal fusion techniques. The best recognition accuracy of 97.00% was achieved for the proposed decision level fusion scheme.

The current works are summarized in Table 1.

## 3. Proposed Method

### 3.1. Hand Skeletal Data

**Figure 1:**
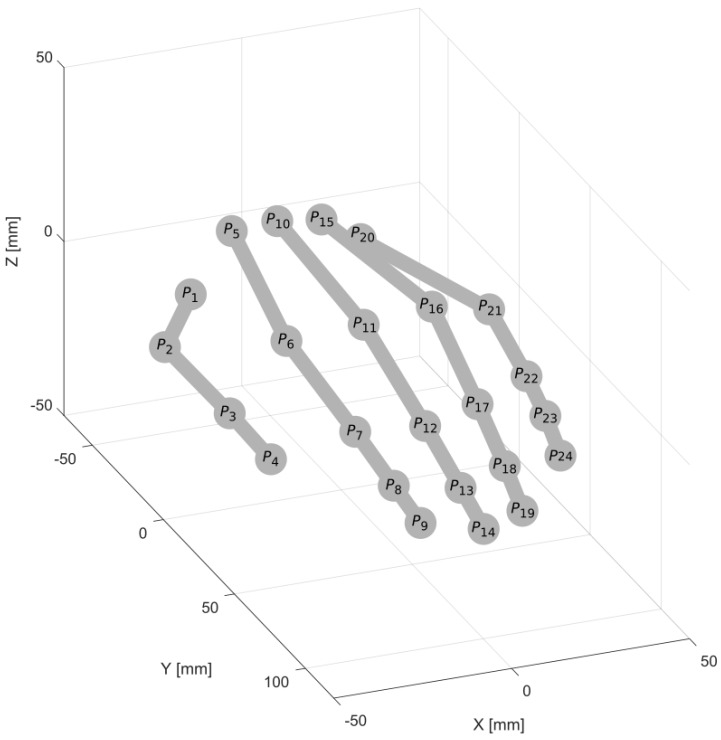
Hand skeletal model.

The skeletal hand model considered in this paper is shown in Figure 1.

It consists of bones visualized in the form of straight line sections and connections between them (joints) depicted as numbered balls. There are four kinds of bones in this model: (i) four metacarpals (between joints $P_{5}$–$P_{6}$, $P_{10}$–$P_{11}$, $P_{15}$–$P_{16}$, $P_{20}$–$P_{21}$), (ii) five proximal phalanges ($P_{1}$–$P_{2}$, $P_{6}$–$P_{7}$, $P_{11}$–$P_{12}$, $P_{16}$–$P_{17}$, $P_{21}$–$P_{22}$), (iii) five intermediate phalanges ($P_{2}$–$P_{3}$, $P_{7}$–$P_{8}$, $P_{12}$–$P_{13}$, $P_{17}$–$P_{18}$, $P_{22}$–$P_{23}$), and (iv) five distal phalanges ($P_{3}$–$P_{4}$, $P_{8}$–$P_{9}$, $P_{13}$–$P_{14}$, $P_{18}$–$P_{19}$, $P_{23}$–$P_{24}$).

In a contactless way, such a model can be acquired directly using LMC released in 2012 [23] or Intel RealSense device released in 2015 [24] and embedded in some laptop models. A simplified version of the model, sufficient to determine the feature vector proposed in this paper, can be also obtained using Softkinetic DepthSense 325 camera along with the Close Interaction Library [25]. LMC has been recently evaluated [26], but there are no many publications about RealSense due to its recent release. It is expected that in the near future these devices and the supplied software will be further improved to allow for reliable skeletal hand tracking.

### 3.2. Feature Vector

**Figure 2:**
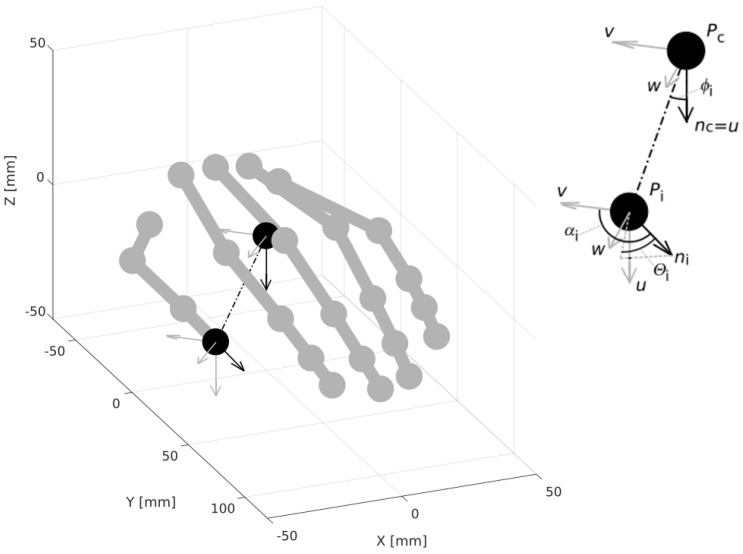
Feature vector construction.

The proposed feature vector encodes the relative differences between vectors associated with the pointing directions of the fingers and the palm normal. Let $P_{c}$ be the center of the palm, $n_{c}$ normal to the palm at point $P_{c}$, $P_{i}$ the end of the *i*-th finger, and $n_{i}$ the vector pointed by that finger (Figure 2).

The relative position of vectors $n_{c}$ and $n_{i}$ can be unambiguously described giving four values determined from the Formulas (1)–(4) [27]:

(1) $$\alpha_{i}=acos\left(v_{i}\cdot n_{i}\right)$$

(2) $$\phi_{i}=acos\left(u\cdot \frac{d_{i}}{|d_{i}|}\right)$$

(3) $$\Theta_{i}=atan\left(\frac{w_{i}\cdot n_{i}}{u\cdot n_{i}}\right)$$

(4) $$d_{i}=P_{i}-P_{c}$$

where the vectors *u*, $v_{i}$, and $w_{i}$ define the so-called Darboux frame [28]:

(5) $$u=n_{c}$$

(6) $$v_{i}=\frac{d_{i}}{|d_{i}|}\times u$$

(7) $$w_{i}=u\times v_{i}$$

and · indicates the scalar and ×—vector products. Since the $d_{i}$ vectors depend on the size of the hand, they have been omitted. The feature vector consists of 15 values calculated for individual fingers using the Formulas (1)–(3):

(8) $$V=[\alpha_{1},\phi_{1},\Theta_{1},\alpha_{2},\phi_{2},\Theta_{2},\alpha_{3},\phi_{3},\Theta_{3},\alpha_{4},\phi_{4},\Theta_{4},\alpha_{5},\phi_{5},\Theta_{5}]$$

In the case of LMC, the palm center, the palm normal and the pointing directions of the fingers are returned along with the skeletal data. For other devices, they can be derived from the skeletal data using the Formulas (9)–(11) (see Figure 1):

(9) $$P_{c}=\frac{1}{10}\sum_{j\in J}P_{j}$$

(10) $$n_{c}=\vec{P_{15}P_{16}}\times \vec{P_{5}P_{6}}$$

(11) $$n_{i}=\vec{P_{3+5(i-1)}P_{4+5(i-1)}}$$

where J={1,2,5,6,10,11,15,16,20,21}.

### 3.3. Classification

The following classification methods have been tested: decision trees (DT) [29], linear and quadratic discriminants (LD and QD) [30,31], support vector machines with linear, quadratic, cubic and Gaussian kernel function (SVM Lin/Quad/Cub/Gauss) [32,33,34], different version of k-nearest neighbor classifiers (1 NN, 10 NN, 100 NN, 10 NN Cos, 10 NN W) [35,36], different ensemble classifiers, that meld results from many weak learners into one model (Ens Boost/Bag/RUS/SubD/kNN) [37,38,39,40] and fast approximate nearest neighbors with randomized kd-trees (FLANN) [41]. A detailed list of tested classifiers with their initial parameters is provided in Table 2.

## 4. Experiments

### 4.1. Datasets

Two datasets were considered.

#### 4.1.1. Dataset 1: Authors’ Own Dataset

**Figure 3:**
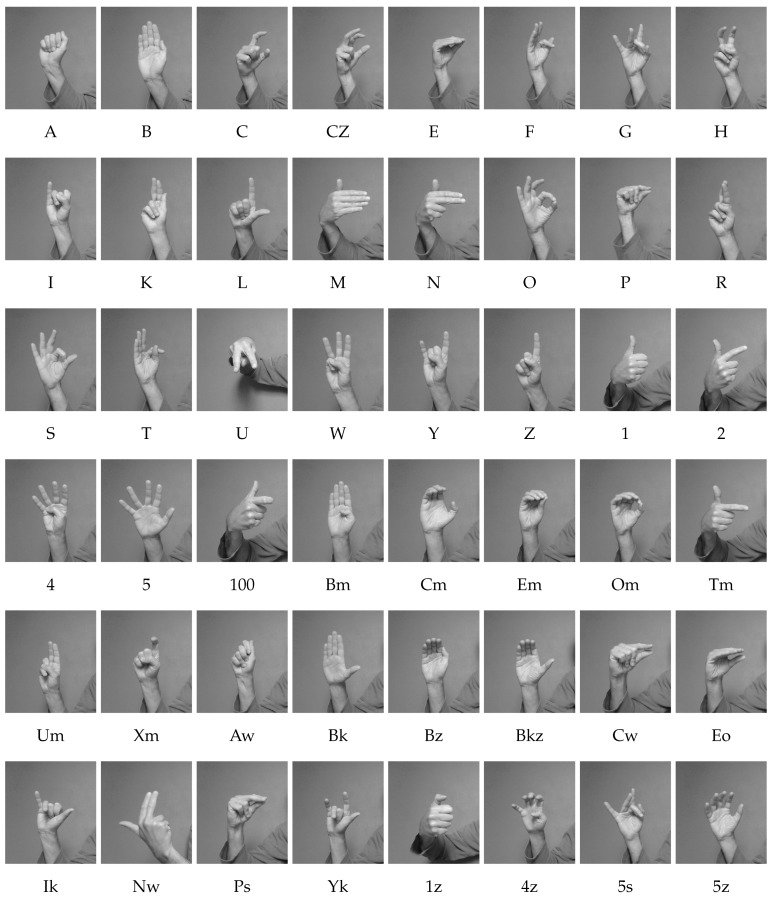
Static handshapes, occurring in Polish Finger Alphabet and Polish Sign Language.

Forty-eight static handshapes, occurring in Polish Finger Alphabet (PFA) and Polish Sign Language (PSL) were considered (Figure 3) [25].

The gestures were recorded in two configurations: (i) LMC lies horizontally on the table (configuration user-sensor); (ii) the sensor is attached to the monitor and directed towards the signer (configuration user-user). In the configuration (i), two variants were additionally considered: (a) gestures are made with fixed hand orientation (like in PFA); (b) spatial hand orientation changes in a wide range (like in PSL). In the configuration (i) variant (a) five people, designated hereinafter A, B, C, D, and E, participated in the recordings. In other cases, the gestures of person A were recorded. Gestures were shown by each person 500 times. During the data collection, visual feedback was provided, and when an abnormal or incomplete skeleton was observed, the process was repeated to ensure that 500 correct shapes were registered for each class. Incorrect data was observed for approximately 5% of frames. It was also noticed that the device works better when the whole hand with very visible fingers is presented first and then slowly changes to the desired shape.

#### 4.1.2. Dataset 2: Microsoft Kinect and Leap Motion Dataset

In order to evaluate the method for more users and to make a comparative analysis, the database provided in the work [3] was used. The database contains the recordings of 10 letters from ASL, performed 10 times by 14 people and acquired by jointly calibrated LMC and depth sensor.

### 4.2. Results

The results of 10-fold cross-validation for the dataset 1 are shown in Table 3, Table 4 and Table 5.

For LMC lying on the table (configuration (i)) the best recognition rates (≥99.5%) were for SVM, kNN, Ens Bag, Ens Sub kNN and FLANN, wherein the results obtained under large variation in hand’s rotation (variant (b)) were only slightly worse. For configuration (ii), the results are better. This configuration seems to be more natural for a user accustomed to showing gestures to another person.

However, the results of the leave-one-subject-out cross-validation experiment, shown in Table 6 and Table A1, are much worse for all considered classification methods. The best recognition rates (≥50.0%) were for: LD, SVM Lin, Ens Bag.

The performances of the individual gestures are strongly dependent on the user, and the training set consisting of four people is not sufficiently representative to correctly classify the gestures of the fifth, unknown person.

The results obtained for dataset 2, and shown in Table 7, Table 8 and Table A2, confirm that when the training set consists of more users, the discrepancy between 10-fold cross-validation and leave-one-subject-out cross-validation is significantly lower. However, it should be mentioned that in this case, the number of recognized classes is much smaller.

For the dataset 2 and 10-fold CV, the best results (≥88.0%) were for SVM Gauss, kNN 1, kNN W, Ens Bag and Ens Sub kNN, whereas for leave-one-subject-out cross-validation the best results (≥88.0%) were for kNN1, kNN W, Ens Sub kNN.

Because for the most demanding case (Table 6) the best results were obtained for SVM Lin and Ens Bag—the parameters of these two classifiers were further analyzed (see Table 9 and Table 10).

The SVM classifier is by nature binary. It classifies instances into one of the two classes. However, it can be turned into a multinomial classifier by two different strategies: one-vs-one and one-vs-all. In one-vs-one, a single classifier for each pair of classes is trained. The decision is made by applying all trained classifiers to an unseen sample and a voting scheme. The class that has been recognized most times is selected. In one-vs-all, a single classifier per class is trained. The samples of that class are positive samples, and all other samples are negatives. The decision is made by applying all trained classifiers to an unseen sample and selecting the one with the highest confidence score.

**Figure 4:**
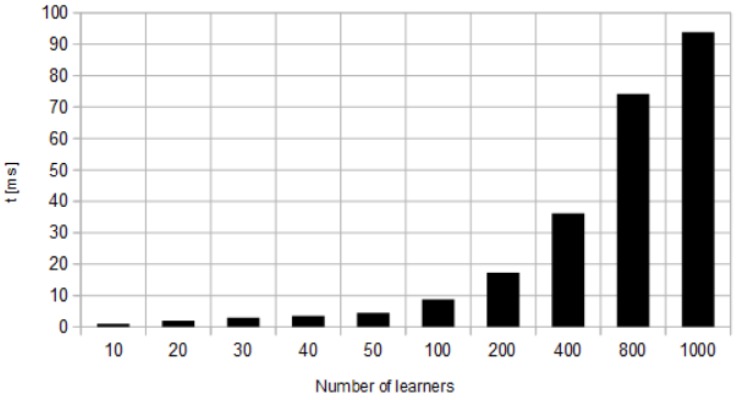
Response time for Ens Bag classifier for different number of learners.

In SVM Lin classifier, the change of the multiclass method from one-vs-one to one-vs-all leads to decrease in the recognition accuracy. For Ens Bag classifier, the recognition accuracy increases with the number of learners, but the response time increases as well (see Figure 4).

The experiment was stopped when the response time reached 100 ms, i.e., the value at which the typical user will notice the delay [42].

In Table 8 the best results were obtained for 1 NN, 10 NN W, and Ens Sub kNN. The FLANN version of the kNN classifier turned out to be the fastest one. Therefore, a further analysis of kNN classifiers has been carried out.

In Table 11, the nearest neighbor classifier 1 NN with brute-force search in the dataset was compared with the FLANN version with a different number of the randomized trees.

As should be expected, the results obtained for the exact version are slightly better than for the classifier, which finds the approximate nearest neighbor. However, if we compare the processing times, the FLANN version is over 400 times faster. Therefore, this classifier is a particularly attractive choice in practical applications.

An experiment was also carried out to check whether the late fusion of classifiers, at the decision level of individual models, leads to improved recognition accuracy. A simple method was used, in which every classifier votes for a given class. According to [43], simple unweighted majority voting is usually the best voting scheme. All possible combinations of classifiers were tested. The best result of leave-one-subject-out cross-validation on dataset 1, 56.7%, was obtained when the outputs of the classifiers LD, QD, SVM Lin, Ens Boost, Ens Bag were fused. The result is better than the best result obtained for a single classifier by 4.4%. However, the fusion of classifiers leads to a decrease in the individual classes recognition. The voting deteriorates the prediction in classes F, I, Xm, Yk.

### 4.3. Computational Efficiency

The average response times of the individual classifiers are shown in Table 12.

Together with the average time needed for data acquisition and feature vectors calculation, which is equal to 6 ms, they do not exceed 100 ms, so the typical user will not notice the time delay between presentation of the given gesture and the predicted response of the system [42]. However, all experiments were carried out on a fairly powerful workstation, equipped with a 2.71 GHz processor, 32 GB of RAM and a fast SSD. For less-efficient systems, e.g., mobile or embedded devices, the preferred choice is FLANN or DT. Moreover, in the case of FLANN classifier, the randomized trees can be searched in parallel.

### 4.4. Comparative Analysis

According to the authors’ knowledge, the only database of static hand skeletal data available on the Internet for which comparative analysis can be carried out is Dataset 2 [3]. Table 13 compares the recognition accuracy obtained for this database.

The first row quotes the results obtained for LMC, without additional data from the Kinect sensor. The proposed feature vector allows obtaining better results even with the same classifier (SVM).

## 5. Conclusions

Handshape recognition based on its skeleton becomes an important research problem because there are more and more new devices on the market that enable the acquisition of such data. In this paper:

- A feature vector was proposed, which describes the relative differences between the pointing directions of individual fingers and the hand normal vector.
- A demanding dataset containing 48 hand shapes, shown 500 times by five persons in two different sensor placement, has been prepared and made available [44].
- The registered data has been used to perform classification. Seventeen known and popular classification methods have been tested.
- For classifiers SVM Lin and Ens Bag, given the best recognition accuracies, an analysis of the impact of their parameters on the obtained results was carried out.
- It was found that the weaker result for leave-one-person-out validation may be caused by individual character of performances of individual gestures, a difficult dataset, containing as many as 48 classes, among which there are very similar shapes, and imperfections of the LMC, which in the case of individual fingers occlusions tries predict their position and spatial orientation. It is worth mentioning that other works on static handshape recognition, cited in the literature, concern a smaller number of simpler gestures.
- The proposed feature vector allows obtaining better results.
- It was determined experimentally that although late fusion improves the results, it causes the deterioration of recognition efficiency in some classes, which in some applications may be undesirable.

To recognize complicated handshapes occurring in the sign languages, a feature vector invariant to translation, rotation, and scale, which is sensitive to the subtle differences in shape, is needed. The proposed feature vector is inspired by research on local point cloud descriptors [27]. Angular features, describing the mutual position of two vectors normal to the cloud surface, are used there to form a representation of the local geometry. Such a descriptor is sensitive to subtle differences in shape [45]. In our proposition, the fingertips and the palm center are treated as a point cloud, and the finger directions and the palm’s normal are used instead of the surface normals. It is also worth noting that the proposed feature vector is invariant to position, orientation, and scale. This is not always the case in the literature, where the features depending on the hand size or orientation are used. This invariance is particularly important in the case of sign language, where unlike in the finger alphabet, the hand’s position and orientation are not fixed. An interesting proposition of an invariant feature vector was proposed in [3] and enhanced in [19]. In Section 4.4, it was compared to our proposal.

Analysis of the confusion matrices obtained for the dataset 1 shows that the most commonly confused shapes are: B-Bm, C-100, N-Nw, S-F, T-O, Z-Xm, Tm-100, Bz-Cm and 4z-Cm. In fact, these are very similar shapes (see Figure 3). In adverse lighting conditions, when they are viewed from some distance or from the side, they can be confused even by a person. When sequences of handshapes, corresponding to fingerspelled words, are recognized, disambiguation can be achieved by using the temporal context. However, this is not always possible because often fingerspelling is used to convey difficult names, foreign words or proper names. If the similar shapes are discarded from the dataset 1, leave-one-subject-out cross-validation gives recognition efficiencies of about 80%.

The proposed system is fast and requires no special background or specific lighting. One of the reasons for the weaker results of leave-one-person-out validation is the imperfection of a sensor, that does not cope well with fingers occlusions. Therefore, as part of further work, the processing of point clouds registered with two calibrated sensors is considered in order to obtain more accurate and reliable skeletal data. Further work will also include recognition of letter sequences and integration of the presented solution with the sign language recognition system.

## Figures and Tables

**Table 1:** Recent works on handshape recognition using skeletal data.

Work	Sign	Sign	Users	Device	Method	Accuracy	Data
	Type	Vocabulary				[%]	Available
[3]	static	10 letters ASL	14	LMC	SVM	80.86	Yes
				LMC + Kinect		91.28	
[4]	static	26 letters ASL	2	LMC	kNN	72.78	No
					SVM	79.83	
[5]	static	28 letters ArSL	1	LMC	NB	98.3	No
					MP	99.1	
[6]	dynamic	50 sign ArSL	2	LMC	MP	88	No
[7]	static	10 digits ASL	8	2 LMs	HMM	93.14	No
[8]	static	24 letters ASL	1	LMC	DT + GA	82.71	No
[9]	static	5 simple shapes	?	LMC	BSR	?	No
[10]	static	10 digits ISL	4	LMC	MP	100	No
[11]	static	28 letters ArSL	20	LMC + Kinect	SVM	86	No
[12]	dynamic	25 sign ISL	10	LMC + Kinect	CHMM	90.80	Yes
[13]	static	28 letters ArSL	4	LMC + Kinect	kNN	100	No
[14]	static	26 letters SIBI	1	LMC	RB-BGANN + GA	93.8	No
[15]	static	20 letters ASL	50	LMC, RealSense	SVM	60–100	No
[16]	static	26 letters ASL	10	LMC	kNN	70–100	No
		10 digits ASL				95–100	
[17]	static	44 letters ThSL	?	LMC	DT	72.83	No
[18]	static	28 letters ArSL	1	2 LMs	LDA	97.7	No
[19]	static	10 hand shapes	13	LMC	SVM	99.42	No
[20]	dynamic	28 signs and	10	LMC	SVM + BLSTM	63.57	No
		28 words ISL					
[21]	static	8 hand shapes	20	LMC	kNN	95	Yes
[22]	static	10 hand shapes	14	LMC + Kinect		97	No

**Table 2:** Tested classifiers and their parameters.

Classifier	Parameter	Value
DT	Maximum number of splits	100
	Split criterion	Gini’s diversity index
LD	Covariance structure	Full
QD	Covariance structure	Full
SVM Lin/Quad/Cub/Gauss	Kernel function	Linear/Quadratic/Cubic/Gaussian
	Box constraint level	1
	Multiclass method	One-vs-one
1 NN/10 NN/100 NN	Number of neighbors	1 /10/100
	Distance metric	Euclidean
10 NN Cos	Number of neighbors	10
	Distance metric	Cosine
10 NN W	Number of neighbors	10
	Distance metric	Euclidean
	Distance weight	Squared inverse
Ens Boost/Bag/RUS	Ensemble method	Boosted/bagged/random subspace trees
	Learner type	Decision tree
	Number of learners	30
Ens Sub D/Sub kNN	Ensemble method	Subspace
	Learner type	Discriminant/1 NN
	Number of learners	30
	Subspace dimension	8
FLANN	Number of neighbors	1
	Number of trees	8
	Number of times the trees	128
	should be recursively traversed	

**Table 3:** 10-fold cross-validation results for dataset 1, configuration (i), variant (a).

Classifier	DT	LD	QD	SVM	SVM	SVM	SVM	1 NN	10 NN
				Lin	Quad	Cub	Gauss		
Recognition rate [%]	81.2	72.8	99.7	99.5	100.0	100.0	100.0	100.0	99.9
**Classifier**	**100 NN**	**10 NN**	**10 NN**	**Ens**	**Ens**	**Ens**	**Ens**	**Ens**	**FLANN**
		**Cos**	**W**	**Boost**	**Bag**	**Sub D**	**Sub kNN**	**RUS**	
Recognition rate [%]	99.2	99.9	100.0	64.2	100.0	69.9	100.0	39.9	100.0

**Table 4:** 10-fold cross-validation results for dataset 1, configuration (i), variant (b).

Classifier	DT	LD	QD	SVM	SVM	SVM	SVM	1 NN	10 NN
				Lin	Quad	Cub	Gauss		
Recognition rate [%]	83.1	78.7	97.2	96.7	99.4	99.7	99.1	99.8	98.5
**Classifier**	**100 NN**	**10 NN**	**10 NN**	**Ens**	**Ens**	**Ens**	**Ens**	**Ens**	**FLANN**
		**Cos**	**W**	**Boost**	**Bag**	**Sub D**	**Sub kNN**	**RUS**	
Recognition rate [%]	88.9	98.5	99.5	67.1	99.7	77.3	99.8	35.8	99.7

**Table 5:** 10-fold cross-validation results for dataset 1, configuration (ii).

Classifier	DT	LD	QD	SVM	SVM	SVM	SVM	1 NN	10 NN
				Lin	Quad	Cub	Gauss		
Recognition rate [%]	100.0	100.0	100.0	100.0	100.0	100.0	100.0	100.0	100.0
**Classifier**	**100 NN**	**10 NN**	**10 NN**	**Ens**	**Ens**	**Ens**	**Ens**	**Ens**	**FLANN**
		**Cos**	**W**	**Boost**	**Bag**	**Sub D**	**Sub kNN**	**RUS**	
Recognition rate [%]	100.0	100.0	100.0	100.0	100.0	100.0	100.0	43.8	100.0

**Table 6:** Leave-one-subject-out cross-validation results for dataset 1, configuration (i), variant (1).

Classifier	DT	LD	QD	SVM	SVM	SVM	SVM	1 NN	10 NN
				Lin	Quad	Cub	Gauss		
Recognition rate [%]	41.4	50.9	42.5	52.3	49.1	46.9	14.0	48.6	48.6
**Classifier**	**100 NN**	**10 NN**	**10 NN**	**Ens**	**Ens**	**Ens**	**Ens**	**Ens**	**FLANN**
		**Cos**	**W**	**Boost**	**Bag**	**Sub D**	**Sub kNN**	**RUS**	
Recognition rate [%]	48.9	47.9	48.7	43.3	50.8	46.8	49.9	28.7	47.6

**Table 7:** 10-fold cross-validation results for dataset 2.

Classifier	DT	LD	QD	SVM	SVM	SVM	SVM	1 NN	10 NN
				Lin	Quad	Cub	Gauss		
Recognition rate [%]	87.6	84.5	86.4	87.6	86.2	84.1	88.4	88.6	88.0
**Classifier**	**100 NN**	**10 NN**	**10 NN**	**Ens**	**Ens**	**Ens**	**Ens**	**Ens**	**FLANN**
		**Cos**	**W**	**Boost**	**Bag**	**Sub D**	**Sub kNN**	**RUS**	
Recognition rate [%]	82.6	87.9	89.1	87.8	88.9	85.1	88.5	86.9	85.9

**Table 8:** Leave-one-subject-out cross-validation results for the dataset 2.

Classifier	DT	LD	QD	SVM	SVM	SVM	SVM	1 NN	10 NN
				Lin	Quad	Cub	Gauss		
Recognition rate [%]	86.2	84.2	87.5	87.6	86.4	82.3	86.7	89.2	85.5
**Classifier**	**100 NN**	**10 NN**	**10 NN**	**Ens**	**Ens**	**Ens**	**Ens**	**Ens**	**FLANN**
		**Cos**	**W**	**Boost**	**Bag**	**Sub D**	**Sub kNN**	**RUS**	
Recognition rate [%]	82.4	85.6	89.6	87.0	87.7	84.1	89.3	86.4	85.4

**Table 9:** Support Vector Machines classifier with linear kernel function performance when the multiclass method was changed from one-vs-one to one-vs-all.

Training	Testing	SVM Lin
B, C, D, E	A	36.8
A, C, D, E	B	21.1
A, B, D, E	C	47.8
A, B, C, E	D	52.0
A, B, C, D	E	46.6
Avg		40.8

**Table 10:** Ens Bag performance for a different number of learners.

Training	Testing	10	20	30	40	50	100	200	400	800	1000	2000
B, C, D, E	A	44.6	47.4	39.6	46.8	45.3	46.8	46.9	46.1	45.4	45.9	46.3
A, C, D, E	B	40.1	40.3	40.5	38.0	38.6	37.0	38.8	39.2	40.0	41.4	38.7
A, B, D, E	C	53.2	56.6	59.0	56.1	55.8	53.9	58.6	58.3	57.4	57.7	58.0
A, B, C, E	D	54.8	54.6	56.1	54.8	58.5	57.9	57.0	57.4	57.9	58.1	57.9
A, B, C, D	E	58.7	60.0	58.7	60.6	58.6	63.4	60.5	61.2	62.5	63.0	61.7
Avg		50.4	51.8	50.8	51.3	51.3	51.8	52.4	52.4	52.6	53.2	52.5

**Table 11:** 1 NN vs. FLANN with a different number of trees (given in parenthesis).

Training	Testing	1 NN	FLANN (1)	FLANN (2)	FLANN (4)	FLANN (8)	FLANN (16)	FLANN (32)
B, C, D, E	A	43.1	38.0	41.2	39.5	40.4	41.1	40.4
A, C, D, E	B	32.3	31.1	30.9	33.6	33.6	33.0	33.6
A, B, D, E	C	60.6	55.8	56.5	57.4	56.1	57.0	56.4
A, B, C, E	D	52.3	52.1	51.3	51.3	51.1	51.5	51.2
A, B, C, D	E	54.5	55.1	56.6	56.5	55.7	55.5	55.6
Avg		48.6	46.4	47.3	47.6	47.4	47.6	47.4

**Table 12:** Average response times of the individual classifiers.

Classifier	DT	LD	QD	SVM	SVM	SVM	SVM	1 NN	10 NN
				Lin	Quad	Cub	Gauss		
Response time [ms]	0.07	0.46	0.42	26.73	31.98	30.12	64.87	24.15	26.64
**Classifier**	**100 NN**	**10 NN**	**10 NN**	**Ens**	**Ens**	**Ens**	**Ens**	**Ens**	**FLANN**
		**Cos**	**W**	**Boost**	**Bag**	**Sub D**	**Sub kNN**	**RUS**	
Response time [ms]	29.24	22.50	23.14	3.22	2.95	9.3	47.65	4.14	0.06

**Table 13:** 10-fold cross validation results of different methods obtained for the Dataset 2.

Lp	Reference	Features	Method	Recognition Rate
1	[3]	Fingertips distances, angles and elevations	Multiclass SVM	80.9%
2	[19]	Fingertips Tip distance	Multiclass SVM	81.1%
3	This paper	As described in Section 3.2	SVM Lin	87.6%
4	This paper	As described in Section 3.2	10NN W	89.1%

## Appendix A. Results of Leave-One-Person-Out Cross-Validation

**Table A1:** Leave-one-subject-out cross-validation results for dataset 1, configuration (i), variant (1).

Training	Testing	DT	LD	QD	SVM	SVM	SVM	SVM	1 NN	10 NN
					Lin	Quad	Cub	Gauss		
B, C, D, E	A	37.4	51.3	32.1	46.1	41.0	38.8	4.0	43.1	43.3
A, C, D, E	B	31.2	37.6	29.6	33.0	32.9	31.0	5.6	32.3	31.7
A, B, D, E	C	47.9	54.7	41.6	57.8	57.5	55.4	21.8	60.6	61.2
A, B, C, E	D	41.6	53.8	51.1	58.6	54.0	51.7	18.7	52.3	51.3
A, B, C, D	E	48.9	57.1	58.1	66.1	60.1	57.4	19.8	54.5	55.5
Avg		41.4	50.9	42.5	52.3	49.1	46.9	14.0	48.6	48.6
**Training**	**Testing**	**100 NN**	**10 NN**	**10 NN**	**Ens**	**Ens**	**Ens**	**Ens**	**Ens**	**FLANN**
			**Cos**	**W**	**Boost**	**Bag**	**Sub D**	**Sub kNN**	**RUS**	
B, C, D, E	A	45.0	44.3	43.2	38.7	39.6	50.4	42.2	27.6	40.9
A, C, D, E	B	32.6	32.7	31.8	37.6	40.5	31.3	35.9	16.4	33.8
A, B, D, E	C	60.4	55.7	61.6	46.6	59.0	51.0	60.4	35.3	56.5
A, B, C, E	D	48.9	51.7	51.3	45.2	56.1	44.8	52.0	30.1	50.8
A, B, C, D	E	57.7	55.3	55.4	48.5	58.7	56.5	59.0	33.9	56.1
Avg		48.9	47.9	48.7	43.3	50.8	46.8	49.9	28.7	47.6

**Table A2:** Leave-one-subject-out cross-validation results for the dataset 2.

Training	Testing	DT	LD	QD	SVM	SVM	SVM	SVM	1 NN	10 NN
					Lin	Quad	Cub	Gauss		
2–14	1	85.0	76.0	77.0	77.0	77.0	71.0	79.0	95.0	74.0
1, 3–14	2	89.0	87.0	91.0	90.0	90.0	91.0	91.0	89.0	90.0
1–2, 4–14	3	81.0	88.0	91.0	91.0	84.0	77.0	83.0	82.0	84.0
1–3, 5–14	4	89.0	92.0	97.0	96.0	95.0	87.0	97.0	94.0	94.0
1–4, 6–14	5	90.0	90.0	92.0	91.0	91.0	87.0	92.0	86.0	92.0
1–5, 7–14	6	91.0	92.0	93.0	92.0	92.0	89.0	93.0	93.0	92.0
1–6, 8–14	7	87.0	85.0	88.0	88.0	86.0	80.0	87.0	84.0	89.0
1–7, 9–14	8	86.0	90.0	92.0	92.0	87.0	90.0	93.0	91.0	92.0
1–8, 10–14	9	86.0	84.0	87.0	87.0	84.0	78.0	87.0	88.0	86.0
1–9, 11–14	10	84.0	77.0	89.0	89.0	89.0	86.0	81.0	86.0	85.0
1–10, 12–14	11	79.0	72.0	74.0	80.0	80.0	75.0	73.0	76.0	74.0
1–11, 13–14	12	90.0	94.0	100.0	100.0	100.0	96.0	100.0	95.0	97.0
1–12, 14	13	85.0	76.0	77.0	77.0	77.0	73.0	79.0	95.0	74.0
1–13	14	85.0	76.0	77.0	77.0	77.0	72.0	79.0	95.0	74.0
Avg		86.2	84.2	87.5	87.6	86.4	82.3	86.7	89.2	85.5
**Training**	**Testing**	**100 NN**	**10 NN**	**10 NN**	**Ens**	**Ens**	**Ens**	**Ens**	**Ens**	**FLANN**
			**Cos**	**W**	**Boost**	**Bag**	**Sub D**	**Sub kNN**	**RUS**	
2–14	1	73.0	76.0	95.0	77.0	80.0	76.0	95.0	74.0	95.0
1, 3–14	2	89.0	90.0	91.0	91.0	89.0	87.0	90.0	89.0	89.0
1–2, 4–14	3	88.0	84.0	82.0	84.0	83.0	88.0	82.0	90.0	82.0
1–3, 5–14	4	89.0	94.0	95.0	97.0	97.0	92.0	94.0	92.0	94.0
1–4, 6–14	5	90.0	91.0	89.0	92.0	92.0	91.0	86.0	92.0	80.0
1–5, 7–14	6	88.0	92.0	93.0	93.0	93.0	92.0	93.0	93.0	87.0
1–6, 8–14	7	82.0	89.0	87.0	88.0	86.0	88.0	83.0	88.0	78.0
1–7, 9–14	8	88.0	91.0	90.0	92.0	90.0	91.0	91.0	91.0	91.0
1–8, 10–14	9	86.0	85.0	86.0	87.0	88.0	84.0	88.0	87.0	88.0
1–9, 11–14	10	75.0	84.0	84.0	85.0	89.0	76.0	86.0	89.0	86.0
1-10, 12-14	11	74.0	74.0	78.0	78.0	79.0	72.0	75.0	79.0	69.0
1–11, 13–14	12	85.0	96.0	94.0	100.0	100.0	89.0	97.0	98.0	95.0
1–12, 14	13	73.0	76.0	95.0	77.0	82.0	76.0	95.0	74.0	70.0
1–13	14	73.0	76.0	95.0	77.0	80.0	76.0	95.0	74.0	91.0
Avg		82.4	85.6	89.6	87.0	87.7	84.1	89.3	86.4	85.4
